# Targeting the Mitochondria-Proteostasis Axis to Delay Aging

**DOI:** 10.3389/fcell.2021.656201

**Published:** 2021-03-11

**Authors:** Andreas Zimmermann, Corina Madreiter-Sokolowski, Sarah Stryeck, Mahmoud Abdellatif

**Affiliations:** ^1^Institute of Molecular Biosciences, University of Graz, Graz, Austria; ^2^Field of Excellence BioHealth – University of Graz, Graz, Austria; ^3^Gottfried Schatz Research Center, Medical University of Graz, Graz, Austria; ^4^Institute of Interactive Systems and Data Science, Graz University of Technology, Graz, Austria; ^5^Department of Cardiology, Medical University of Graz, Graz, Austria; ^6^Metabolomics and Cell Biology Platforms, Institut Gustave Roussy, Villejuif, France; ^7^Centre de Recherche des Cordeliers, Equipe Labellisée Par la Ligue Contre le Cancer, Université de Paris, Sorbonne Université, INSERM U1138, Institut Universitaire de France, Paris, France

**Keywords:** aging, proteostasis, mitochondria, autophagy, anti-aging targets

## Abstract

Human life expectancy continues to grow globally, and so does the prevalence of age-related chronic diseases, causing a huge medical and economic burden on society. Effective therapeutic options for these disorders are scarce, and even if available, are typically limited to a single comorbidity in a multifaceted dysfunction that inevitably affects all organ systems. Thus, novel therapies that target fundamental processes of aging itself are desperately needed. In this article, we summarize current strategies that successfully delay aging and related diseases by targeting mitochondria and protein homeostasis. In particular, we focus on autophagy, as a fundamental proteostatic process that is intimately linked to mitochondrial quality control. We present genetic and pharmacological interventions that effectively extend health- and life-span by acting on specific mitochondrial and pro-autophagic molecular targets. In the end, we delve into the crosstalk between autophagy and mitochondria, in what we refer to as the mitochondria-proteostasis axis, and explore the prospect of targeting this crosstalk to harness maximal therapeutic potential of anti-aging interventions.

## Introduction

Human lifespan is continuously rising, which not only reweaves the social fabric of our society but also entails huge economic and medical burden due to the unprecedented prevalence of chronic diseases. Therapeutic options to delay the onset of age-related maladies are scarce, which is often further complicated by associated comorbidities. For instance, in the US, 62% percent of those over 65 years suffer from more than one chronic condition (Jaul and Barron, 2017). Therefore, therapies which target fundamental processes of aging itself rather than treating each comorbidity as a separate clinical entity are desperately needed.

Aging manifests in a continuous decline of organismal homeostasis. Accumulating defects on the cellular level can result in cellular dysfunction that impairs normal physiology. This damage can be of extrinsic origin e.g., mutagenic radiation and toxins, or intracellular origin, like harmful reactive oxygen species (ROS) generated by defective mitochondrial respiration (Bornstein et al., 2020), advanced glycation end products (Semba et al., 2010) or the accumulation of toxic protein aggregates (Hipp et al., 2019). The consequences of such harm are particularly devastating to post-mitotic, fully differentiated cells with low cellular turnover rates, such as neuronal cells and cardiomyocytes (Ernst et al., 2014; Eschenhagen et al., 2017). To mitigate the detrimental effects of extrinsic and intrinsic noxa, eukaryotic cells have developed various protective mechanisms. One such mechanism is proteostasis, a collective term for a network of protein quality control and degradation pathways that ensure the normal expression, folding and turnover of proteins. During aging, proteostasis, like other cellular functions, suffer from a progressive decline, which renders the body more vulnerable to damage and age-related diseases (David, 2012; Wiersma et al., 2016). In particular, neurodegenerative (Hipp et al., 2019) and cardiovascular diseases (Wiersma et al., 2016; Abdellatif et al., 2020) are increasingly attributed to such age-related decline in proteostasis.

In terms of anti-aging interventions, (macro)-autophagy, a major catabolic pathway for macromolecules and even whole organelles, has proven to be a pharmacologically pliable mechanism to improve cellular proteostasis. Accumulating evidence suggests that mitochondrial function and proteostasis, in particular autophagy, are highly intertwined (Andréasson et al., 2019). This imposes new constraints on anti-aging therapies because the positive outcome of selectively targeting one of these processes (e.g., autophagy activation) might be limited if the other process is negatively affected (e.g., excessive mitochondrial clearance). Even the most efficient anti-aging intervention known to date, which is caloric restriction without malnutrition (CR), fails when components of the mitochondria-proteostasis axis are defective (Zhou et al., 2019). At the same time, behavioral or pharmacological interventions, such as CR mimetics (CRMs) that replicate molecular signatures of CR, are likely to have the best efficacy, when they are able to improve both proteostasis as well as mitochondrial function.

Here, we summarize current strategies that successfully delay aging and aging-associated diseases by targeting autophagy, and/or mitochondrial homeostasis. In addition, we explore options to specifically target the mitochondria-autophagy crosstalk to harness maximal anti-aging potential of this axis.

## Targeting Autophagy to Delay Aging

**FIGURE 1:**
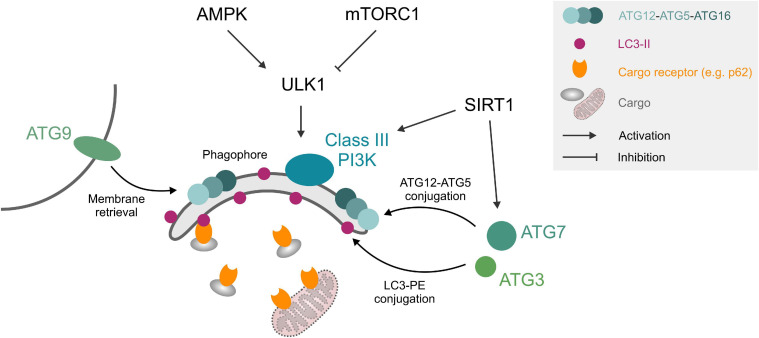
Autophagy core machinery. Upstream nutrient/growth factor signaling and cellular energy status regulate mTORC1 and AMPK kinases, respectively, which in turn inhibit or activate ULK1 through different phosphorylation sites. ULK1 phosphorylates components of the phagophore formation machinery, consisting of a class III PI3K complex, ATG12-ATG5-ATG16 complex, ATG9, and LC3-II. LC3-II can interact with cargo (e.g., protein aggregates) through cargo receptors such as SQSTM1/p62 or organelles like mitochondria through mitochondrial surface markers. ATG3 and ATG7 mediate ATG12-ATG5 and LC3-PE conjugation. SIRT1 deacetylates and activates several ATG proteins. See text for details. Abbreviations: LC3-II, microtubule-associated protein light chain 3 (lipidated form); PE, phosphatidylethanolamine; PI3K, phosphatidylinositol 3 kinase; ULK1, Unc-51-like kinase 1.

The autophagic machinery consists of core autophagy-related (ATG) proteins, which mediate the recognition of cargo structures and the recruitment of membranes for the engulfment of cellular material into double-membraned vesicles, called autophagosomes. Autophagy initiation can be triggered by upstream signaling pathways such as starvation or stress-signaling, which relay environmental changes to the UNC-51-like kinase 1 (ULK1) complex through a network of kinases and phosphatases. Upon ULK1 activation, double membrane isolation membranes, called phagophores, form under the participation of class III phosphatidylinositol 3 kinase (PI3K) complex, the membrane recruitment factor ATG9 and the tripartite ATG12-ATG5-ATG16 protein complex. The expanding phagophore is stabilized by the small, membrane-anchored protein microtubule-associated protein light chain 3 (LC3), which also mediates the interaction with cargo receptors that decorate cellular material designated for degradation (Figure 1). After the growing phagophore closes around cellular cargo, the resulting autophagosomes fuse with lysosomes and form autolysosomes, where the autophagic cargo is degraded and recycled (Yin et al., 2016). Proteostasis through this general autophagic degradation pathway (also termed “macro”-autophagy) is complemented by several specialized subroutines, e.g., chaperone-mediated autophagy or autophagic degradation of mitochondria, called mitophagy.

Basal autophagy is essential for the maintenance of normal body function. Mice with whole-body knockout of the autophagy core machinery proteins, like ATG5 and ATG7, die perinatally (Kuma et al., 2017), while conditional tissue-specific disruption, for instance in neurons or cardiomyocytes, leads to premature organ damage in the form of neurodegeneration or dilated cardiomyopathy, respectively (Hara et al., 2006; Taneike et al., 2010). In humans, brain biopsies indicate that core ATG genes such as *ATG5*, *ATG7* (Lipinski et al., 2010), and *BECLIN 1* (Shibata et al., 2006) are downregulated during physiological aging, except in healthy centenarians, who reportedly show elevated serum levels of the PI3K complex subunit Beclin 1 compared to younger individuals (Emanuele et al., 2014). Consistently, patients with Alzheimer’s disease or mild cognitive impairment show reduced serum levels of ATG5 (Castellazzi et al., 2019). While these human observations should be interpreted with caution, because ATG expression levels *per se* do not necessary correlate with autophagic activity (Klionsky et al., 2021), it is generally accepted that autophagy levels decrease with age and under many pathological conditions (Levine and Kroemer, 2008). In support of this notion, artificial activation of autophagy by ubiquitously overexpressing ATG5 extends health and lifespan in mice (Pyo et al., 2013). Prolonged lifespan was also observed upon neuronal overexpression of ATG8a in Drosophila (Simonsen et al., 2008). Hence, activating autophagy may not only counteract aging-associated pathologies but also slow down the aging process in general and therefore constitutes an attractive target for pharmacological interventions.

Autophagy can be modulated either by (i) targeting upstream signaling pathways, in particular insulin/IGF-1 receptor activity and AMP/ATP ratio, (ii) directly targeting proteins of the autophagic machinery, or (iii) improving the clearance of autophagosomes and their cargo by enhancing lysosomal function (Table 1). In general, a distinction between interventions that delay aging *through* autophagy induction and interventions that do so *in association with*, i.e., epiphenomenal, autophagy induction should be made. This is particularly important when studying CR mimetics or other bioactive compounds that target processes upstream of autophagy. For instance, the mTOR inhibitor rapamycin induces autophagy and extends Drosophila lifespan but fails to do so when ATG5 is downregulated. At the same time, the lifespan extension is also lost when the other branch of mTOR downstream signaling, namely translation control via S6 kinase and 4EBP is shunted (Bjedov et al., 2010). Here, laboratory model organisms such as *Caenorhabditis elegans* and *Drosophila melanogaster* as well as an increasing number of conditional and tissue-specific knockout mouse models are indispensable tools for a relatively fast and easy assessment of the causal role of autophagy or alternative processes.

**TABLE 1:** Defined molecular targets that delay aging by acting on the mitochondria-proteostasis axis.

Mechanism	Target	Intervention	Organism/system	Outcome	References
Upstream activation of autophagy	↑ AMPK	- Salicylates - Metformin	*- C. elegans* - Mice	Lifespan and healthspan extension	Martin-Montalvo et al., 2013; Shamalnasab et al., 2018; Bharath et al., 2020
	↓ Insulin/IGF-1	*-* Genetic mutation (*daf-2*) - Diazoxide	*- C. elegans -* Rats	Lifespan extension	Melendez, 2003; Tóth et al., 2008; Meng et al., 2018
	↑ SIRT1	- Resveratrol - NAD^+^ precursors	- *C. elegans* - Mice	- Lifespan extension - Delayed cardiac aging	Barger et al., 2008; Morselli et al., 2010a, b; Abdellatif et al., 2021
	↓ mTORC1	- Rapamycin	*- C. elegans -* Drosophila - Mice	Lifespan extension	Bjedov et al., 2010; Robida-Stubbs et al., 2012; Neff et al., 2013
Direct activation of autophagy machinery proteins	↑ ULK1	- BL-918 molecule	- Mice	Neuroprotection	Ouyang et al., 2018
	↑ Beclin 1	- Genetic mutation (Beclin 1^*F*121A^) - Tat-Beclin 1 peptide	- Mice	- Extension of lifespan and healthspan (↓ renal, cardiac and oncological pathologies) - Ameliorated cardiac and memory performance	Shirakabe et al., 2016; Fernández et al., 2018; Sun et al., 2018; Glatigny et al., 2019
Lysosomal clearance of autophagic cargo	↑ TFEB	- Genetic overexpression (HLH-30) - Small-molecule TFEB activators - Trehalose	- *C. elegans* - Mice	- Lifespan extension - Hepatoprotection - Attenuated vascular aging	LaRocca et al., 2012; Lapierre et al., 2013; Wang et al., 2017
Mitochondrial biogenesis	↑ PGC-1α	- PPARγ agonists (GW1929) - Cell-specific genetic overexpression	- Human dopaminergic neurons - Mice (skeletal myocytes) - Mice (cardiomyocytes)	- Neuroprotection - Extension of median lifespan in females and maximal lifespan in males and enhanced endurance capacity and protection from age-related sarcopenia, but aggravated age-related bone loss - Accentuated cardiac aging and shorter lifespan with higher overexpression, but cardioprotection with moderate overexpression	Mäkelä et al., 2016; Garcia et al., 2018; Whitehead et al., 2018; Zhu et al., 2019; Yang S. et al., 2020
Mitochondrial fission	↑ DRP1	- Genetic upregulation - Mitochondrial division inhibitor-1	- Drosophila (midlife) - Mice (skeletal muscle) - Mice (intravenous injection)	- Lifespan and healthspan extension - Insulin resistance and impaired muscle development - Protection against cardiac ischemia-reperfusion injury	Ong et al., 2010; Jheng et al., 2012; Touvier et al., 2015; Rana et al., 2017
Mitochondrial respiration	↓ ETC	- Genetic disruption (Mit mutants) - Cytochrome c oxidase moderate inhibitor	- *C. elegans* - Mice	- Lifespan extension - Healthspan extension, including reduced visceral fat and enhanced glucose homeostasis	Rea et al., 2007; Tavallaie et al., 2020

### Targeting Upstream Signaling Pathways Regulating Autophagy

Under nutrient-rich conditions, autophagy is repressed, while nutrient depletion or a high AMP/ATP ratio increases autophagic activity. Interestingly, these molecular signatures coincide with those elicited by CR and other anti-aging fasting regimes (Madeo et al., 2019). In fact, autophagy activation is a common hallmark among nutritional anti-aging interventions (Madeo et al., 2015), although the causal involvement of this activation in the context of CR and fasting remains surprisingly poorly investigated and has mainly been investigated in *C. elegans* mutants that only exhibit CR-like responses (Hansen et al., 2008). Nevertheless, autophagy is essential for the lifespan extending effects of AMP-activated kinase (AMPK) stimulation by salicylic acid derivatives (Shamalnasab et al., 2018). Other AMPK agonists, like metformin have been shown to counteract aging-related inflammation (“inflammaging”) processes through autophagy activation (Bharath et al., 2020). Similarly, lifespan extension due to reduced insulin/insulin-like growth factor (IGF-1) receptor signaling in *C. elegans daf-2* mutants requires functional autophagy (Melendez, 2003; Tóth et al., 2008) and the downstream effector of repressed insulin/IGF-1 signaling, the FOXO transcription factor DAF-16, promotes the expression of autophagy genes (Murphy et al., 2003). Pharmacological modulation of insulin/IGF-1 signaling, in particular by diazoxide has been proposed as a therapeutic avenue against cancer (Klement and Fink, 2016). Indeed, diazoxide reportedly restores autophagy in an osteoarthritis rat model and the beneficial effects were blocked by co-administration of the autophagy inhibitor 3-methyladenine (Meng et al., 2018). Another branch of CR downstream effects is regulated by the nicotinamide adenine dinucleotide (NAD^+^)-dependent deacetylase SIRT1, which directly deacetylates and thereby activates ATG proteins like Beclin 1, LC3, ATG5, and ATG7 (Sacitharan et al., 2020). SIRT1-deficient mice show early perinatal mortality accompanied by elevated acetylation of autophagy factors in heart, brain and liver tissue (Lee et al., 2008). In contrast, SIRT1 activation through CR, NAD^+^ supplementation or the polyphenol resveratrol delays cardiac aging (Abdellatif et al., 2021) and extends lifespan in an autophagy-dependent manner (Morselli et al., 2010a, b). In sum, there is a case to be made for autophagy as one of the main effectors of the modulation of nutrient/growth factor signaling.

### Targeting the Autophagy Machinery Proteins

Autophagy initiation is controlled by ULK1, which is regulated by the upstream kinases AMPK and mTOR complex 1 and in turn, activates other autophagy initiation factors like Beclin 1. Pharmacological activation of ULK1 with the structure-based design molecule BL-918 induces autophagy and has protective effects in a Parkinson’s disease mouse model. This protection is lost when the autophagy blocker 3-methyladenine is co-administered (Ouyang et al., 2018). The ULK1 downstream target Beclin 1 is a regulator of the Beclin 1/VPS34 lipid kinase and, under basal conditions, is kept in an inactive state by a physical interaction with Bcl-2. Under nutrient depletion or stress conditions, this interaction is lost and Beclin 1 can engage with VPS34 to stimulate autophagy initiation. Disrupting this interaction through a Beclin 1^*F*121A^ point mutation increases autophagic activity and extends lifespan in mice (Fernández et al., 2018). Chemical probes that disrupt Beclin 1/Bcl-2 interaction have been discovered but are yet to be tested for their ability to extend lifespan *in vivo* (Chiang et al., 2018). Beclin 1 is also inhibited by interaction with Rubicon (Matsunaga et al., 2009), a protein that accumulates during aging (Nakamura et al., 2019). Rubicon depletion leads to lifespan extension in Drosophila (Nakamura et al., 2019), but to date, no pharmacological inhibitors have been identified. That said, the autophagy-promoting function of Beclin 1 can be mimicked by administration of a modified peptide corresponding to amino acids 267–284 of the protein (termed “Tat-Beclin 1 peptide”) *in vivo* (Shoji-Kawata et al., 2013). Importantly, autophagy activation by Tat-Beclin 1 improves memory performance in aged mice (Glatigny et al., 2019) and protects from heart failure in mice plagued with risk factors, albeit at a young age (Shirakabe et al., 2016; Abdellatif et al., 2018; Sun et al., 2018).

The small membrane-bound protein LC3 does not only provide a membrane anchor for other components of the autophagic machinery but can also directly interact with autophagic cargo or adapter proteins such as sequestosome 1 (SQSTM1/p62). Recently, small molecules have been developed that can promote the interaction of LC3 with specific target proteins, e.g., the Huntington’s disease-associated PolyQ protein huntingtin to mediate their autophagic degradation (Li et al., 2019). This strategy also works in the other direction, as demonstrated for a recently discovered small molecule that can disrupt the interaction of the malaria parasite factor UIS3 with LC3 (Setua et al., 2020). This modulation of interaction of specific target proteins with the autophagic machinery is an exciting therapeutic avenue, and more so as an increasing number of proteins has been shown to form aggregates during aging, which in turn may drive other proteotoxic events such as amyloid beta aggregation (Groh et al., 2017).

### Targeting Lysosomal Function

The degradation of autophagic cargo requires healthy lysosomes. Indeed, blocking lysosomal function by inhibiting lysosomal acidification by bafilomycin A1 or neutralizing lysosomal pH by chloroquine is routinely used to measure the accumulation of autophagosomes experimentally (Klionsky et al., 2021). Lysosomal biogenesis, which constitutes an important factor in the efficacy of autophagy-inducing interventions (Di Malta et al., 2019), is regulated by several transcription factors, most prominently transcription factor EB (TFEB). TFEB activators such as trehalose (Rusmini et al., 2019), 3,4-dimethoxychalcone (Chen et al., 2019) as well as cytosolic Ca^2+^ levels (Medina et al., 2015) stimulate autophagic activity. Overexpression of the TFEB homolog HLH-30 is sufficient to extend *C. elegans* lifespan with concomitant autophagy activation (Lapierre et al., 2013), and small-molecule TFEB activators act hepatoprotective in mice and lifespan-extending in nematodes (Wang et al., 2017). TFEB expression declines with age, at least in human and murine immune cells, due to reduced activity of the translation initiation factor eIF5A (Zhang et al., 2019). This factor is activated by a unique post-translational modification, called hypusination, which requires the polyamine spermidine as a substrate. Intriguingly, spermidine levels also decline during aging, suggesting that reduced TFEB translation through impaired eIF5A hypusination might underlie the aging-associated loss of autophagic activity (Madeo et al., 2018; Zhang et al., 2019). This hypothesis is further corroborated by the fact that spermidine supplementation extends lifespan in various model organisms and protects against age-related cardiac decline in mice in an autophagy-dependent fashion (Eisenberg et al., 2009, 2016).

## Targeting Mitochondria to Delay Aging

Mitochondrial dysfunction is a hallmark of the aging process (López-Otín et al., 2013; Bornstein et al., 2020). Consistently, genetic mutations that dysregulate mitochondrial function clearly associate with accelerated aging phenotypes and increased susceptibility to disease (Bornstein et al., 2020). However, mitochondrial function does not necessarily show a linear decline during the course of aging. In fact, some reports claim a compensatory increase in mitochondrial activity during middle age, and only later on with advanced age that mitochondrial dysfunction arises (Pugh et al., 2013; Baker and Peleg, 2017). Considering such biphasic alteration of mitochondrial activity with age, the timing of mitochondrial manipulation might be a crucial issue in applying mitochondria-directed interventions for the treatment of age-related disorders. This timing aspect is further complicated by the fact that mitochondrial function, especially the activity of the mitochondrial electron transport chain (ETC), is tightly linked to ROS generation, which if produced at high levels can lead to mitochondrial DNA and proteins damage, thereby exacerbating cellular aging and toxicity (Madreiter-Sokolowski et al., 2018).

Structurally, mitochondria rely on a highly dynamic and interconnected network stretching throughout the whole cell. As such, functional mitochondria are constantly regulated through an extensive bidirectional communication with other cellular components, including the endoplasmic reticulum (ER) (Simmen and Herrera-Cruz, 2018), the plasma membrane (Westermann, 2015), endosomes (Das et al., 2016) and lysosomes (Wong et al., 2018). This communication can be mediated by Ca^2+^ ions, which strongly affect mitochondrial function by boosting the activity of Ca^2+^-dependent dehydrogenases of the Krebs cycle and, thereby, the efficacy of the ETC (Denton et al., 1975). Again, this beneficial effect can transition into toxicity, as overwhelming levels of mitochondrial Ca^2+^ might also induce cell death (Celsi et al., 2009), a mechanism reported for several age-related diseases (Calvo-Rodriguez et al., 2020). Consequently, fine-tuning of mitochondrial activity to secure sufficient energy supply, while avoiding exaggerated Ca^2+^ or ROS accumulation, seems essential in order to exploit mitochondria as a target to counteract aging.

In sum, in contrast to autophagy induction, where multiple proteins within the same pathway represent pliable molecular targets, choosing specific targets within the vast mitochondrial network and accurately modulate them to counteract aging (e.g., timing, dosage, duration, etc.) is more challenging (Rea et al., 2007). That said, available evidence suggests that mitochondria can be targeted to successfully delay aging through manipulating either of the following mitochondrial aspects: (i) biogenesis, (ii) dynamics, or (iii) metabolism (Table 1).

### Targeting Mitochondrial Biogenesis

The peroxisome proliferator-activated receptor γ (PPARγ) coactivator α (PGC-1α) is a master regulator of mitochondrial biogenesis, which has been poised as a potential target to antagonize age-related mitochondrial decline. In fact, several studies have demonstrated that health-promoting interventions, like exercise and CR, associate with PGC-1α activation (Kupr and Handschin, 2015), suggesting that it might be genetically or pharmacologically targeted to counteract aging and related disease. Indeed, PPARγ agonists, which are used to treat type-2-diabetes, were shown to elicit neuroprotective effects by a PGC-1α-mediated increase of mitochondrial biogenesis (Mäkelä et al., 2016). Directly targeting PGC-1α by adenovirus-assisted co-expression also rescued loss of dopaminergic neurons upon expression of the PD-associated α-synuclein A53T mutant protein (Zheng et al., 2010). Furthermore, PGC-1α overexpression in skeletal muscle improved mitochondrial content and rejuvenated the molecular signature of aged mice, which exhibited a significant, albeit modest, extension of median lifespan in females and maximal lifespan in males (Garcia et al., 2018). In another study, constitutive overexpression of PGC-1α in skeletal muscle cells recapitulates some features of exercise-mediated anti-aging effects, yet hippocampal neurogenesis which is typically improved by exercise remained unaffected (Karlsson et al., 2019). Besides, functional evaluation of skeletal muscles in aged mice with PGC-1α-overexpressing revealed enhanced endurance capacity and protection from age-related sarcopenia, at least in males. However, these benefits were associated with aggravated age-related trabecular bone loss (Yang S. et al., 2020). In the heart, PGC-1α overexpression, specifically in cardiac myocytes, improved mitochondrial function and cardiac performance; however, these effects were inflected with age, leading to an accentuated cardiac aging phenotype and shorter lifespan (Zhu et al., 2019). In contrast, another report suggested that when moderately overexpressed PGC-1α exerts cardioprotective effects also in aged mice (Whitehead et al., 2018), indicating that fine-tuning of PGC-1α activity is a crucial aspect to consider if the anti-aging potential of PGC-1α is to be harnessed for the development of novel therapeutics.

### Targeting Mitochondrial Dynamics

Mitochondria undergo dynamic changes in their morphology through active fusion and fission to cope with different cellular cues. In this process, the cytosolic dynamin-related protein 1 (DRP1) is considered a key player orchestrating mitochondrial fission, which is required for mitochondrial division, multiplication as well as budding off damaged segments for targeted degradation. Since DRP1 expression is reduced in aged cells, it was proposed as a potential target to counteract age-induced alterations in the mitochondrial network architecture (Mai et al., 2010). In support of this notion, short-term promotion of mitochondrial fission in midlife through DRP1 upregulation prolongs lifespan and healthspan in Drosophila (Rana et al., 2017). Pharmacological approaches to target mitochondrial fission by selectively inhibiting DRP1 were also successfully employed against ischemia/reperfusion injury (Ong et al., 2010) and in cell models of PD (Qi et al., 2013). In stark contrast, embryonic overexpression of DRP1 in the skeletal muscle of mice causes increased mitochondrial fragmentation and dysfunction, and impaired muscle development (Jheng et al., 2012; Touvier et al., 2015), thus indicating that the outcomes of targeting DRP1 and mitochondrial fission depend on the timing, duration and organism involved.

With regard to mitochondrial fusion, it is primarily mediated by two sets of proteins; mitofusin 1 and 2 (MFN1 and MFN2, respectively), as well as optic atrophy 1 (OPA1), which are responsible for the fusion of outer and inner mitochondrial membranes, respectively (Dorn, 2019). Mitochondrial fusion allows for direct metabolite exchange, improved ATP production, and enhanced stress resistance as it dilutes the influence of damaged mitochondria when they join a larger healthy network. In fact, caloric restriction and a myriad of other pro-longevity pathways require mitochondrial fusion to extend lifespan, at least in *C. elegans* (Chaudhari and Kipreos, 2017). However, it is important to note that increasing mitochondrial fusion *per se* is not sufficient to promote longevity, which indicate that targeting mitochondrial fusion has to be done in the context of promoting the dynamic balance between fusion and fission. In line with this idea, mice with a triple cardiac knockout of MFN1, MFN2, and DRP1 live longer than fusion-defective (MFN1/MFN2 double knockout) or fission-defective (DRP1 knockout) mice. However, such complete ablation of mitochondrial dynamics in Mfn1/Mfn2/Drp1 triple knockouts leads later on to impaired mitochondrial quality control, accelerated senescence, and cardiac dysfunction (Song et al., 2017). Taken together, the salutary benefits of targeting mitochondrial fusion or fission seem to be restricted to late-in-life interventions aimed to restore mitochondrial dynamics, rather than tipping off the balance in favor of fusion or fission.

### Targeting Mitochondrial Metabolism

Although the primary function of mitochondria is to generate energy equivalents, moderate reduction of mitochondrial ATP synthesis through reversible pharmacological inhibition of the mitochondrial cytochrome c oxidase (CcO) extends healthspan in mice (Tavallaie et al., 2020). Along similar lines, mutant *C. elegans* with disrupted ETC genes (Mit mutants) exhibit extended lifespan (Rea et al., 2007). However, by titrating the degree of mitochondrial ETC inhibition, these perplexing observations were reconciled, as lifespan extension appeared to be biphasic; i.e., moderate ETC inhibition was associated with lifespan extension, yet as the level of inhibition increased lifespan became shorter. Interestingly, global levels of ROS did not show any correlation with lifespan, suggesting that this effect cannot be attributed to differences in oxidative stress (Rea et al., 2007). The principal concept of a potential positive effect of antioxidants has been also challenged by the finding that small levels of ROS boost the development of antioxidant defense mechanisms, thereby positively affecting health- and lifespan (Ristow, 2014). In support of this finding, the three well-known antioxidants N-acetylcysteine, ascorbic acid (vitamin C) and resveratrol extended lifespan of *C. elegans* at low concentrations, but caused a reduced lifespan at high concentrations (Desjardins et al., 2017). This dosage-dependent effect might also explain the controversial outcomes of clinical trials testing the impact of antioxidant supplementation on the prevention of age-related diseases like cardiovascular and oncological disorders (Lee et al., 2005; Cook et al., 2007). Thus, the usage of antioxidant supplements is questionable and does not seem to positively affect the process of aging. Alternately, interventions targeted to moderate mitochondrial metabolism and energy production might be more promising.

## The Crosstalk Between Mitochondria and Autophagy

Although both mitochondria- and autophagy-targeted interventions effectively delay aging, it remains unclear to what extent these overlap, and whether their simultaneous administration would result in any additional therapeutic value. A growing body of evidence suggests that, at least downstream, a crosstalk between these two longevity-promoting strategies exists. On the one hand, multiple steps of autophagy require ATP supply and, thus, functional mitochondria (Plomp et al., 1989; Li et al., 2017). On the other hand, autophagy regulates mitochondrial quantity and quality control through the degradation of damaged and potentially harmful mitochondria in a highly specialized form of autophagy, known as mitophagy (Abdellatif, 2019). Damaged or dysfunctional mitochondria accumulate the sentinel protein PTEN-induced kinase 1 (PINK1) at the surface, which is usually rapidly imported and then degraded in healthy mitochondria. PINK1 dimers phosphorylate ubiquitin moieties at the mitochondrial surface, which recruits the E3 ligase Parkin, leading to increased protein ubiquitination and the tagging of the damaged mitochondria for mitophagic degradation (Sekine and Youle, 2018).

Although it is challenging to specifically target mitophagy pharmacologically, available evidence supports the notion that mitophagy *per se* is significantly involved in counteracting aging and related disease (Chen et al., 2020). For instance, mitophagy stimulation by the natural compound urolithin A improves mitochondrial respiratory capacity, thereby extending lifespan in *C. elegans* and attenuating age-related skeletal muscle dysfunction in rodents (Ryu et al., 2016). Similarly, induced mitophagy in spermidine-treated mice is associated with improved mitochondrial function, delayed aging, and protection against related cardiovascular diseases (Eisenberg et al., 2016; Tyrrell et al., 2020). A recent study in *C. elegans* suggested that mitophagy is a prerequisite for such protective effect of spermidine, at least against age-related neurodegeneration (Yang X. et al., 2020). In line with this notion, neuron-specific overexpression of Parkin, known to promote mitophagy, is sufficient to attenuate age-related proteotoxicity and extends lifespan in *D. melanogaster* (Rana et al., 2013). Meanwhile, global Parkin overexpression counteracts age-related sarcopenia and cardiac dysfunction in mice (Leduc-Gaudet et al., 2019; Gao et al., 2021). In support of a causal role for mitophagy in longevity promotion, impaired mitophagy due to PINK-1, DCT-1 and PDR-1 (the nematode homologs for NIX and Parkin, respectively) knock down in *C. elegans* significantly curtails lifespan extension induced by gold standard regimens for autophagy induction, including caloric restriction (in *eat-2* mutants), and attenuated insulin/IGF-1 signaling (in long-lived *daf-2* mutants) (Palikaras et al., 2015). Besides selective mitophagy, autophagy may regulate general mitochondrial network architecture, as it has been shown that lysosome–mitochondria membrane contact sites mark the location for subsequent mitochondrial fission (Wong et al., 2018).

As opposed to mitophagy, much less is known about the prospect of a reverse regulation of autophagic activity by mitochondria. Only a handful of studies examined the influence of mitochondria on autophagy, notwithstanding that mitochondria are expected to contribute to an energy-dependent process like autophagy (Plomp et al., 1989; Li et al., 2017). Thomas et al. (2018) demonstrated that mitochondrial complex I function does not only affect the cell ability to induce autophagy, but also determines the amplitude and duration of autophagy induction, at least upon mTOR inhibition. Accordingly, increasing mitochondrial energy production through a shift toward OXPHOS enhances autophagy activation by mTOR inhibitors (Thomas et al., 2018). Not only mitochondrial function, but also its morphology might have an impact on autophagic activity and might even go beyond that to determine the outcomes of autophagy activation in aging. In fact, stimulating mitochondrial dynamics have been shown to extend the lifespan of *C. elegans* and Drosophila in an autophagy-dependent manner (Rana et al., 2017; Liu et al., 2020). Meanwhile, increasing mitochondrial permeability renders autophagy activation non-beneficial or even harmful as demonstrated in *C. elegans* that mitochondrial leakage causes excessive mitochondrial clearance, thereby abolishing the lifespan-extending effect of autophagy induction by caloric restriction (Zhou et al., 2019).

Furthermore, mitochondrial metabolism might indirectly regulate autophagy levels through TCA intermediates, in particular citrate, which is the main substrate for cytosolic acetyl-CoA production. Acetyl-CoA is the sole donor for acetylation reactions, which have an important function in the post-translational regulation of ATG protein activity. For instance, deacetylation of ATG5, ATG7, and LC3 by SIRT1 is considered an important step in starvation-induced autophagy (Lee et al., 2008). Other components like ULK1, ATG3, or tubulin have to get hyperacetylated to be active (Geeraert et al., 2010; Lin et al., 2012; Yi et al., 2012). Reducing cytosolic acetyl-CoA availability by inhibiting the mitochondrial citrate carrier activates autophagy in human cells (Mariño et al., 2014), while blocking mitochondrial acetate to acetyl-CoA conversion leads to elevated cytosolic acetyl-CoA production and blocked autophagic flux in yeast (Eisenberg et al., 2014). These results suggest that there is a constant crosstalk between mitochondrial metabolism and the activity of the autophagic machinery through an acetyl-CoA-mediated surveillance system.

Another link through which mitochondria might regulate autophagy is lipid metabolism. First, mitochondria are a source for phagophore membranes during autophagosome biogenesis (Hailey et al., 2010). Second, mitochondria also provide phospholipids, like phosphatidylethanolamine (PE), which gets covalently linked to LC3 by ATG3 and ATG7 during autophagy initiation to form the active, lipidated LC3-II conjugate (Hsu and Shi, 2017; Martens and Fracchiolla, 2020). LC3 family proteins can also interact with the mitochondria-specific lipid cardiolipin, which can act as a biosensor for oxidative damage and an autophagic degradation signal when it translocates to the outer mitochondrial membrane (Antón et al., 2016). Interestingly, mitochondrial cardiolipin content seems to be linked to lysosomal function, at least *in vitro* (Chen et al., 2008; Bartel et al., 2019). However, it is not known if this indirect modulation of mitochondrial cardiolipin also induces mitophagy, as observed during oxidative damage-induced translocation of cardiolipin to the mitochondrial surface.

## Concluding Remarks

**FIGURE 2:**
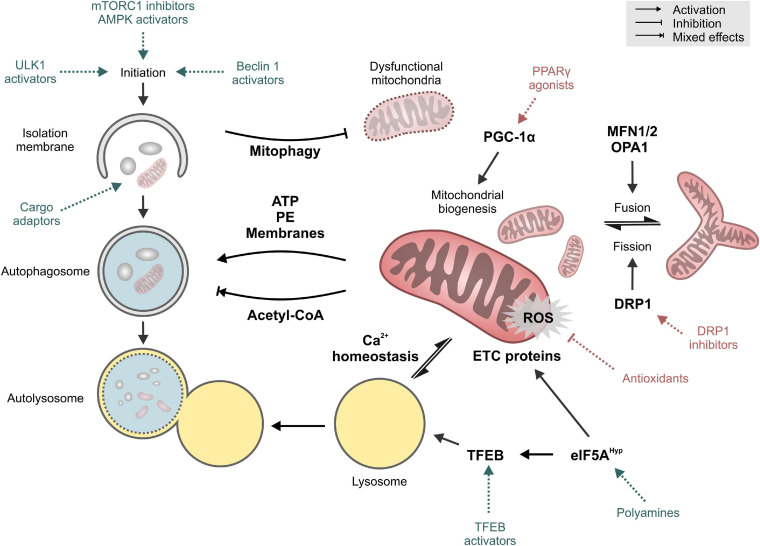
Key targets and mechanisms of crosstalk between autophagy and mitochondria. Pharmacological interventions that mainly target autophagy or mitochondrial function are shown in blue and red, respectively. See text for details. Abbreviations: DRP1, Dynamin-1-like protein; eIF5A^*Hyp*^, hypusinated Eukaryotic translation initiation factor 5A-1; ETC, electron transport chain; MFN1/2, Mitofusin-1/2; OPA1, Optic atrophy protein 1/Dynamin-like 120 kDa protein, mitochondrial; PE, phosphatidyethanolamine; PGC-1α, Peroxisome proliferator-activated receptor gamma coactivator 1-alpha; PPARγ, Peroxisome proliferator-activated receptor gamma; ROS, reactive oxygen species; TFEB, Transcription factor EB; ULK1, Unc-51-like kinase 1.

Mitochondria and autophagy have long been implicated in the process of aging and age-related disorders. More recently though, emerging evidence of a crosstalk between these two longevity targets opens a new avenue for anti-aging interventions. In this perspective, we propose that future research should be aimed toward the identification of causal mechanistic nods linking these two longevity targets (Figure 2). Current observations from model organisms will also need to be verified in mammalian and human tissues. This will be particularly relevant for the organ systems that are mainly constituted of energy-demanding cells, like cardiomyocytes and neurons. Both are long-lived post-mitotic cells that are rich in mitochondria and largely depend on proteostatic mechanisms like autophagy for their quality control. Collectively, the gathered knowledge would guide future development of novel anti-aging therapeutics targeting the mitochondria-proteostasis axis, which might prove more effective against a wide-range of intractable cardiovascular and neurological diseases in the elderly.

## Data Availability Statement

The original contributions presented in the study are included in the article/supplementary material, further inquiries can be directed to the corresponding author.

## Author Contributions

MA and AZ conceived and designed the manuscript. All authors contributed to collecting the literature and writing the first draft of the manuscript and approved the final version of the manuscript for publication.

## Conflict of Interest

The authors declare that the research was conducted in the absence of any commercial or financial relationships that could be construed as a potential conflict of interest.

## Abbreviations

4EBP: eukaryotic translation initiation factor 4E-binding protein
AMPK: AMP activated kinase
ATG: autophagy-related
Bcl-2: apoptosis regulator Bcl-2
CoA: coenzyme A
CR: caloric restriction
CRM: caloric restriction mimetic
DRP1: dynamin-related protein 1
eIF5A: eukaryotic translation initiation factor 5A
ETC: electron transport chain
IGF-1: insulin-like growth factor 1
LC3: microtubule-associated protein 1A/1B-light chain 3
MFN1: mitofusin 1
MFN2: mitofusin 2
mTOR: mechanistic target of rapamycin
OPA1: optic atrophy 1
OXPHOS: oxidative phosphorylation
PD: Parkinson’s disease
PGC-1 α: PPAR γ coactivator α
PI3K: phosphatidylinositol 3 kinase
PINK1: PTEN-induced putative kinase protein 1
PolyQ: polyglutamine tract protein
PPAR γ: peroxisome proliferator-activated receptor
ROS: reactive oxygen species
SIRT1: NAD^+^-dependent protein deacetylase sirtuin-1
TFEB: transcription factor EB
ULK1: UNC-51-like kinase 1
VPS34: phosphatidylinositol 3-kinase VPS34.
